# Electroencephalography derived connectivity informing epilepsy surgical planning: Towards clinical applications and future perspectives

**DOI:** 10.1016/j.nicl.2024.103703

**Published:** 2024-11-10

**Authors:** Giulia Salvatici, Giovanni Pellegrino, Marco Perulli, Alberto Danieli, Paolo Bonanni, Gian Marco Duma

**Affiliations:** a Scientific Institute IRCCS E.Medea, Epilepsy and Clinical Neurophysiology Unit, Conegliano 31015, Italy; b Clinical Neurological Sciences Department, Schulich School of Medicine and Dentistry, Western University, London N6A5C1, Canada; c Fondazione Policlinico Universitario Agostino Gemelli IRCCS, Rome, Italy

**Keywords:** Epilepsy surgery, Drug-resistant epilepsy, EEG, Functional connectivity, Brain dynamics

## Abstract

Epilepsy is one of the most diffused neurological disorders, affecting 50 million people worldwide. Around 30% of patients have drug-resistant epilepsy (DRE), defined as failure of at least two tolerated antiseizure medications (ASMs) to achieve sustained seizure freedom. Brain surgery is an effective therapeutic approach in this group, hinging on the accurate localization of the epileptic focus. The latter task is complex and requires multimodal investigation methods. Epilepsy is also a network disorder and represents one of the best application scenarios of methods leveraging brain functional organization at large scales. Connectivity analysis represents a promising tool for improving surgical assessment, enabling better identification of candidates who could benefit the most from epilepsy surgery. The scalp electroencephalography (EEG) is the most relevant tool to characterize epileptic activity. The EEG has benefited significantly from technological advancement across the last decades. Firstly, electrical source imaging (ESI) allows the reconstruction of electrical activity detected by EEG at the cortex level; secondly, functional connectivity (FC) allows the assessment of functional dependencies across brain areas. The EEG has therefore expanded potential applications in the localization and characterization of the epileptogenic network for surgical planning. As the translation of these methods in clinical practice is little discussed in the literature, we reviewed the investigations using EEG-derived FC. We showed that the FC-informed identification of the epileptic networks improves the localization precision in focal epilepsy. We discussed the heterogeneity in the results and methodology preventing prompt research-to-clinic translation. We finally provided practical suggestions for promoting the applicability of FC-based research in real clinical practice, looking for future research.

## Highlights

• Brain surgery is one of the most effective approaches in drug-resistant epilepsy.
• Electrical source imaging and functional connectivity inform non-invasively the localization of the epileptogenic network.
• The methodological heterogeneity in connectivity application represents a limitation for the clinical translation.
• Investigations on benchmarking connectivity measures and prospective studies may promote applications in clinical practice.
• Future investigation would benefit from the inclusion of personalized methods as *in-silico* simulative approaches.

## 1 Introduction

Epilepsy is one of the most common neurological disorders, with an estimated 52.5 million people affected worldwide and a cost of 119.3 billion dollars per year (Beghi, 2020). Around 30 % of patients have drug-resistant epilepsy (DRE) (Kalilani et al., 2018), defined as failure of at least two tolerated antiseizure medications (ASMs) to achieve sustained seizure freedom (Kwan et al., 2010). Brain surgery is an effective therapeutic approach in this group, with seizure freedom at 1 year of up to 90 % and 60 % in temporal and neocortical epilepsy, respectively (Andrews and Chang, 2020).

Surgery is a local treatment and its success has been linked to the accurate identification of the epileptogenic zone (EZ) (Lüders et al., 2006, Tatum et al., 2018). Nevertheless, recent advancements in neuro-imaging and electrophysiology indicate that epilepsy is a network disorder, with seizures arising from abnormally interconnected regions (epileptic network − EN) (Bernasconi, 2017). A multimodal approach, based on the convergence of electro-clinical, structural and metabolic findings, is generally applied in clinical practice for the identification of the EZ and EN.

Scalp electroencephalography (EEG) has a definite role in this context as it records electrical activity generated by large assemblies of neuronal populations. The EEG is a cost-effective, user-friendly and non-invasive technique employed for about a century in epilepsy to detect excessive and hypersynchronous activity characterizing seizures (Feyissa and Tatum, 2019). The EEG activity is traditionally classified as ictal or interictal, depending on whether it is associated with a seizure or not. The cortical generators of both interictal and ictal discharges, as well as resting state activity, can be identified thanks to modeling techniques comprehensively referred to as electrical source imaging (ESI). The ESI rendered EEG a neuroimaging tool, allowing the accurate identification of brain regions most involved in seizure generation (Pellegrino et al., 2016). Additionally, EEG signals reconstructed over the patient’s brain can be further modeled to infer excitation/inhibition balance (Duma et al., 2024) and functional connections with other regions (Coito et al., 2016, Duma et al., 2021, Pelle et al., 2024). In this scenario, ESI-derived connectivity analysis is a fundamental tool delineating the configuration of the functional cross-talk across regions involved in the ignition and sustaining of the seizures, with high spatio-temporal resolution. Therefore, the application of functional connectivity (FC) has a twofold implication: i) to potentially delineate non-invasively the EN (Pelle et al., 2024); ii) to track the dynamic changes of network organization, unveiling pathophysiological mechanisms to be clinically targeted (Duma et al., 2022, Rosch et al., 2018). EEG-derived functional connectivity is being considered for pre-surgical assessment, but its real clinical value is still debated.

The purpose of this scoping review is to summarize the existing evidence for the application of EEG-derived FC for the pre-surgical study of drug-resistant epilepsy.

The application of ESI and ESI-derived FC metrics is a complex and not standardized process, requiring multiple pre- and post-processing steps. Additionally, the large variability of metrics increases the complexity of a correct choice based on different clinical scenarios. Therefore, to provide some relevant context, we will briefly introduce the principle of FC methodology focusing on the metrics and methodology included in the reviewed papers.

### 1.1 Definition of functional connectivity

Functional connectivity (FC) can be defined as statistical interdependency between the activity (time series) of two neural units (Sporns et al., 2000). A variety of methods are applied to quantify the functional connections, both in the time (time series) and frequency (power and phase after spectral decomposition) domains. The simplest FC metric is based on Pearson correlation (Corr) between the time series of two nodes (He et al., 2019). Some of the most diffused FC metrics based on the EEG power spectrum are Coherence (Coh) and Amplitude envelope correlation (AEC). The Coherence is computed via the cross-correlation of the power spectrum at a specific frequency (Rubinov and Sporns, 2010), whereas Amplitude envelope correlation uses the correlation of the signal envelope (signal amplitude variations over time in a specific frequency range) (Bruns et al., 2000, Hipp et al., 2012). Additional FC metrics use the signal phase instead of the power, as for example phase locking value (PLV) or phase lag index (PLI). The principle of these metrics is that functionally coupled regions show synchronized oscillatory activity resulting in a phase difference close to zero (Lachaux et al., 1999). Finally, the investigation of FC in literature also exploited the general framework of cross–frequency coupling, which allows for studying the interactions between high-frequency amplitude and low-frequency phases (phase-amplitude coupling; PAC) (Samiee and Baillet, 2017).

### 1.2 Effective connectivity

FC metrics do not typically trace the directionality of the information flow across regions. Other methods of so-called effective connectivity (EC), are able to surpass this limitation by estimating the directionality of the interaction between regions (A → B ≠ B → A) (Friston, 2011). Despite the heterogeneity of EC metrics, they are often based on multivariate autoregressive (MVAR) models, which provide an estimation of the activity at a specific time point as a linear combination of the past values of itself and the other variables in the system (other brain regions).

Some of the most diffused MVAR-derived EC metrics are partial directed coherence (PDC) and directed transfer function (DTF), and they are based on frequency analysis (Fasoula et al., 2013, Kuś et al., 2004). The DTF estimates the signal flow from a node *y* to a node *x* , indicating the causal inflow from *y* to *x* relative to the total inflow into *x*. By contrast, the PDC measures the causal information outflow from *y* to *x* relative to the total outflow from *y*. Some studies provided adjusted versions of these metrics, such as adaptive, directed and spectrum-weighted adaptive DTF (aDTF, dDTF, sw-aDTF) or information and renormalized PDC (iPDC, rPDC).

Another measure inferring interaction directionality between brain regions is the mutual information (MI), which describes the amount of information shared between 2 time series (in this context, shared between 2 brain regions/electrodes) (Wang et al., 2014). A final EC method relies on dynamic causal modeling (DCM). In this framework, a number of directional models are hypothesized in advance, and the data is tested to assess which of the models is more likely to explain the recorded activity (Van de Steen et al., 2019).

### 1.3 Basics of network analysis

Connectivity measures are usually stored in an adjacency matrix expressing the magnitude of the mutual relationship between units. While FC results in a symmetrical matrix, not considering the directionality of the information flow (A → B = B → A), EC-derived connectivity matrices are asymmetric. To extract information, the connectivity matrix is treated as a network.

A network is composed of nodes (connected elements) and edges (or links), indicating the connections across pairs of nodes (Rubinov and Sporns, 2010). In the context of EEG-derived connectivity, nodes are typically represented by electrodes or, in the context of ESI, by brain regions (regions of interest; ROIs), whereas edges indicate the intensity of the connections.

#### 1.3.1 Graph theory metrics

The investigation of network configuration contained in the adjacency matrix often involves a mathematical formalization known as graph theory. Common metrics used to characterize node connectivity patterns are **centrality measures** derived from graph theory. A measure of centrality provides information on the importance of a specific node in the information flow across the network. There are several centrality measures, such as Degree, Betweenness, Closeness, or Eigenvector centrality, capturing different aspects of the relevance of a node in a network. Other measures, such as clustering coefficient and local efficiency, inform on the efficiency and the information integration derived from the cross-talk between neighbor areas, in which a specific node represents a central and coordinating element (Fornito et al., 2016). In a figurative scenario, a node with a high degree (number of connections), clustering coefficient and local efficiency may be a fundamental hub to the network architecture, whose removal will influence the structure at the whole network level. Finally, graph measures like small-worldness (SW) offer a broader view distinguishing it from node-specific metrics by focusing on the entire network's connectivity behavior in terms of integration-segregation balance (Rubinov and Sporns, 2010).

The above-presented introduction was meant to provide a general framework helping the reader to navigate through the complexity of the methods and the results presented in the reviewed papers. In the following sections, we will highlight applications of EEG-derived connectivity in the context of epilepsy surgery, evidencing actual limitations and tracing potential solutions for the promotion of the clinical application of a promising method.

## 2 Materials and methods

### 2.1 Literature screening

**Fig. 1:**
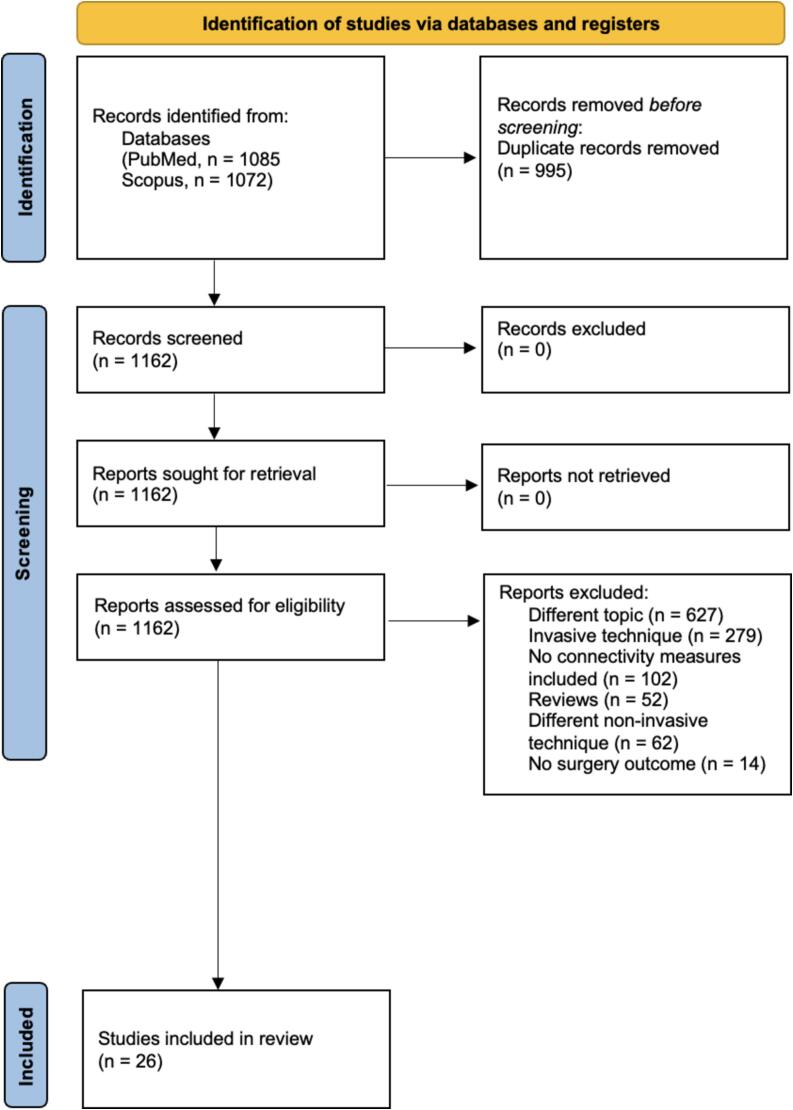
Flow diagram describing the steps necessary to evaluate a scoping review according to the Preferred Reporting Items for Systematic Reviews and Meta-Analyses (PRISMA) criteria*.*

We performed a literature search for peer-reviewed articles reporting results following PRISMA (Preferred Reporting Items for Systematic Reviews and Meta-Analyses) criteria for systematic review. We applied research strings in two online scientific databases, namely PubMed and Scopus, up to March 08, 2024, without region restriction. The full research strings and the number of related papers per string are reported in the Supplementary Material (see Supplementary Materials). Results were screened by S.G. and D.G.M. for inclusion. Initially, 1162 articles were selected, of which 1136 were excluded for the following reasons: 627 papers investigated topics non-related to the review; 279 articles focused on invasive EEG; 102 articles did not have FC measures; 52 articles were reviews; 62 articles did not rely on EEG measurements; 14 articles did not take into account surgical outcomes. The screening process resulted in 26 unique studies from the database search (see Fig. 1 for a summary).

## 3 Results

### 3.1 Characterization of the sample in the studies

A total of 496 patients are reported in 26 studies but at least 77 patients are present in more than one study, leading to 419 unique patients. Age was undefined in 4 papers. About half of the patient’s sample (175 of 390) included pediatric individuals (<18 years old). Sex was undefined in 7 papers. Sex was reported for 300 patients, with about half of the individuals being male (155/300). Twenty out of 26 studies focused on resective surgery with a curative aim. One study reported patients with large or hemispheric etiologies (8/21 ischemic injury, 7/21 Rasmussen encephalitis, 6/21 malformations of cortical development (MCD)). In 19 studies, patients had mesial temporal sclerosis, focal cortical dysplasia, tumors, and more rarely focal sequelae of acquired pathology (stroke, infection, traumatic brain injury). Notably, 4/26 studies reporting a total of 31 patients did not provide information on etiology and MRI findings. In 6/26 studies, surgical treatment was palliative corpus callosotomy. In particular 2 studies for a total of 52 patients focused on infantile spasms syndrome (ISS), an epilepsy encephalopathy with epileptic spasms, hypsarrhythmia on the EEG, and developmental delay in children aged 1–24 months (Zuberi et al., 2022). In this group, most patients (45/52) had corpus callosotomy after failure of medical treatment. Three studies described the effects of callosotomy in patients with Lennox-Gastaut syndrome (LGS). LGS is an epilepsy syndrome with variable etiology characterized by tonic seizures and at least another seizure type, typical generalized EEG features, intellectual disability, and onset before 18 years (Specchio et al., 2022). The studies report 30 unique patients with mixed etiology: 15 (50 %) had normal or unremarkable MRI, and 15 had unilateral (7) or bilateral MCD. Finally, one case reported findings on a bilateral subcortical band heterotopia. Demographic and clinical information of the revised papers are reported in Table 1.

**Table 1:** Demographic and clinical information of the screened articles. Diagnosis abbreviations: DRE, drug-resistant epilepsy; R, right; L, left; TLE, temporal lobe epilepsy; FLE, frontal lobe epilepsy; OLE, orbital lobe epilepsy; PLE, parietal lobe epilepsy; FCD, focal cortical dysplasia; LG, Lennox-Gastaut; I, insular; Surgical intervention abbreviations: P, palliative; R, resective.

**Authors (year of publication)**	**N° of patients**	**Age (years, mean ± standard deviation)**	**Diagnosis**	**Surgical intervention**
Carboni et al. (2019)	19	24.3 ± 11.9	Focal epilepsy	R
Corona et al. (2021)	8	12.5 ± 4.7	DRE	R
Corona et al. (2023)	37	13.8 ± 5.1	DRE	R
Elshoff et al. (2013)	11	9.6 ± 6.9	DRE	R, P (1 hemispherotomy)
Hur and Kim (2015)	27	11.1 ± 4.2	LG syndrome	12 R, 15P (callosotomy)
Hur and Kim (2016)	18	10.8 ± 3.5	LG syndrome	8 R after P (callosotomy), 10P (callosotomy)
Iandolo et al. (2021)	21	11.4 ± 5.7	DRE	R
Li et al. (2016)	10	29.7 ± 11.4	DRE	R
Liang et al. (2017)	14	7.4 ± 4.1	LG syndrome	P(callosotomy)
Liang et al. (2018)	30	8.3 ± 4.6	LG syndrome	P(callosotomy)
Lopes et al. (2020)	15	24.7 ± 13.2	DRE	R
Lu et al. (2012)	10	39.3 ± 12.7	DRE	R
Martinez-Vargas et al. (2017)	3	41, 26 and 64	DRE	R
Matsuhashi et al. (2022)	1	23	DRE	P(callosotomy)
Ntolkeras et al. (2024)	50	11.6 ± 5.7	DRE	R
Pepi et al. (2022)	21	7.9 ± 6.1	DRE	P(hemispherotomy)
Baba et al. (2019)	42	2.3 ± 1.6	Infantile spasms	P(callosotomy)
Sohrabpour et al. (2016)	2	N.A.	PLE and TLE	R
Staljanssens et al. (2017b)	5	37.6 ± 5.5	DRE	R
Staljanssens et al. (2017a)	27	33.9 ± 13.1	FLE (1 R, 1 L), TLE (16 R, 8 L), 1 L OLE	R
Storti et al. (2017)	12	43 ± 13	Focal epilepsy	R
Uda et al. (2021)	10	3.3 ± 2.6	Infantile spasms	P (5 quadrant disconnection, 8 callosotomy, 2 hemispherotomy)
Ueda et al. (2021)	13	0.5 ± 0.55	13 FCD (type Ⅱ, 3 type Ⅰ)	P (posterior quadrantectomy)
Varatharajah et al. (2022)	64	40.5 (range 18–77)	TLE	R
Vespa et al. (2020)	24	15.5 ± 12.1	FLE (9 R, 2 L), FILE (1 R, 1 L), multilobar hemispheric (3 R, 3 L), posterior quadrant (2 R, 1 L), 1 L FTLE	13 R, 11P (6 hemispherotomy, 5 disconnection)
Yao and Li (2021)	2	N.A.	L central gyrus and R OLE	R

### 3.2 EEG data included in the analyses

Ten out of 26 studies computed FC analysis on ictal activity, while the remaining used interictal (with and/or without interictal epileptic discharges, IEDs) EEG. Relevantly, 8 out of 26 articles directly computed FC analyses on sensor space (between EEG electrodes), while the remaining 18 studies employed the ESI method before the FC computation. We identified a large variability of the ESI and FC metrics, which are summarized in Table 2.

**Table 2:** Methodological information of the screened articles. Forward and inverse model abbreviations: LSMAC, locally spherical model with anatomical constraints; LAURA, local auto regressive average; BEM, boundary elementary model; LCMV, linearly constrained minimum variance; ICA, independent component analysis; MUSIC, multiple signal classification; LORETA, low-resolution brain electromagnetic tomography; eLORETA, exact LORETA; FINE, first principle vectors; FDM, finite difference method; taMSP, time adaptive multiple sparse volumetric priors; sLORETA, standardized LORETA; MNE, minimum norm estimate. Connectivity measures abbreviations: PLI, phase lag index; PDC, partial directed coherence; iPDC, information PDC; AEC, amplitude envelope coupling; PLV, phase locking value; rPDC, renormalized PDC; DTF, directed transfer function; dDTF, direct DTF; PAC, phase amplitude coupling; Corr, Pearson correlation; MI, mutual information; aDTF, adaptive DTF; sw-aDTF, spectrum-weighted aDTF; Coh, coherence. Graph theory metrics abbreviations: E, efficiency; BC, betweenness centrality; CC, closeness centrality; EC, eigenvector centrality; GC, global centrality; GE, global efficiency; LE, local efficiency; CPL, characteristic path length; CCoeff, clustering coefficient; LCCoeff, local clustering coefficient; GCCoeff, global clustering coefficient; SW, small worldness; //, method not used.

**Authors (year of publication)**	**N° of electrodes**	**Data**	**Forward and Inverse model**	**Connectivity measures**	**Graph Theory measures**
Carboni et al. (2019)	129/256	Interictal with IEDs	LSMAC and LAURA	iPDC	whole brain, hemispheric and lobar E, hemispheric and lobar connections
Corona et al. (2021)	70	Interictal with and without IEDs	BEM and LCMV	AEC, corr and PLV	BC, CC, degree, EC
Corona et al. (2023)	70	Interictal with and without IEDs	BEM and LCMV	AEC and PLV	BC, CC, Degree, EC
Elshoff et al. (2013)	38–50	Ictal	five-concentric-spheres model and LCMV	DICS and rPDC	//
Hur and Kim (2015)	22	Ictal	BEM and ICA	dDTF	//
Hur and Kim (2016)	21	Ictal	BEM and dipole fitting	PDC and dDTF	//
Iandolo et al. (2021)	25	Interictal without IEDs	BEM and LCMV	AEC	BC, CC, EC, GC
Li et al. (2016)	19–25	Ictal	BEM and MUSIC	PAC	//
Liang et al. (2017)	19	Interictal without IEDs	//	Corr	BC, Degree, CPL, GE, LE and CCoeff
Liang et al. (2018)	19	Interictal without IEDs	//	MI	BC, LCCoeff, CPL, GCCoeff, SW
Lopes et al. (2020)	19	Ictal	BEM and eLORETA	PLV	//
Lu et al. (2012)	21–76	Ictal	BEM and FINE	DTF	//
Martinez-Vargas et al. (2017)	27	Ictal	FDM and taMSP	sw-aDTF	//
Matsuhashi et al. (2022)	16	Interictal with and without IEDs	//	Coh	//
Ntolkeras et al. (2024)	19–25	Interictal with and without IEDs	BEM and LCMV	AEC	//
Pepi et al. (2022)	19	Interictal without IEDs	//	Coh	//
Baba et al. (2019)	19	Interictal with and without IEDs	//	PLI	//
Sohrabpour et al. (2016)	76/64	Ictal (P1) and Interictal (P2)	BEM and sLORETA/MNE	aDTF	//
Staljanssens et al. (2017b)	32–204	Ictal	FDM and LORETA	sw-aDTF	//
Staljanssens et al. (2017a)	27–32	Ictal	FDM and LORETA	sw-aDTF	//
Storti et al. (2017)	256	Interictal with IEDs	BEM and LORETA	aDTF	BC
Uda et al. (2021)	19	Interictal	//	PAC	//
Ueda et al. (2021)	19	Interictal without IEDs	//	PLI	//
Varatharajah et al. (2022)	31	Interictal without IEDs	//	Coh	//
Vespa et al. (2020)	19–29	Ictal and interictal with IEDs	6 layer and LORETA	sw-aDTF	//
Yao and Li (2021)	19	Post-ictal	BEM and sLORETA	sw-aDTF	//

### 3.3 The role of functional connectivity in pre-surgical and post-surgical assessment

**Fig. 2:**
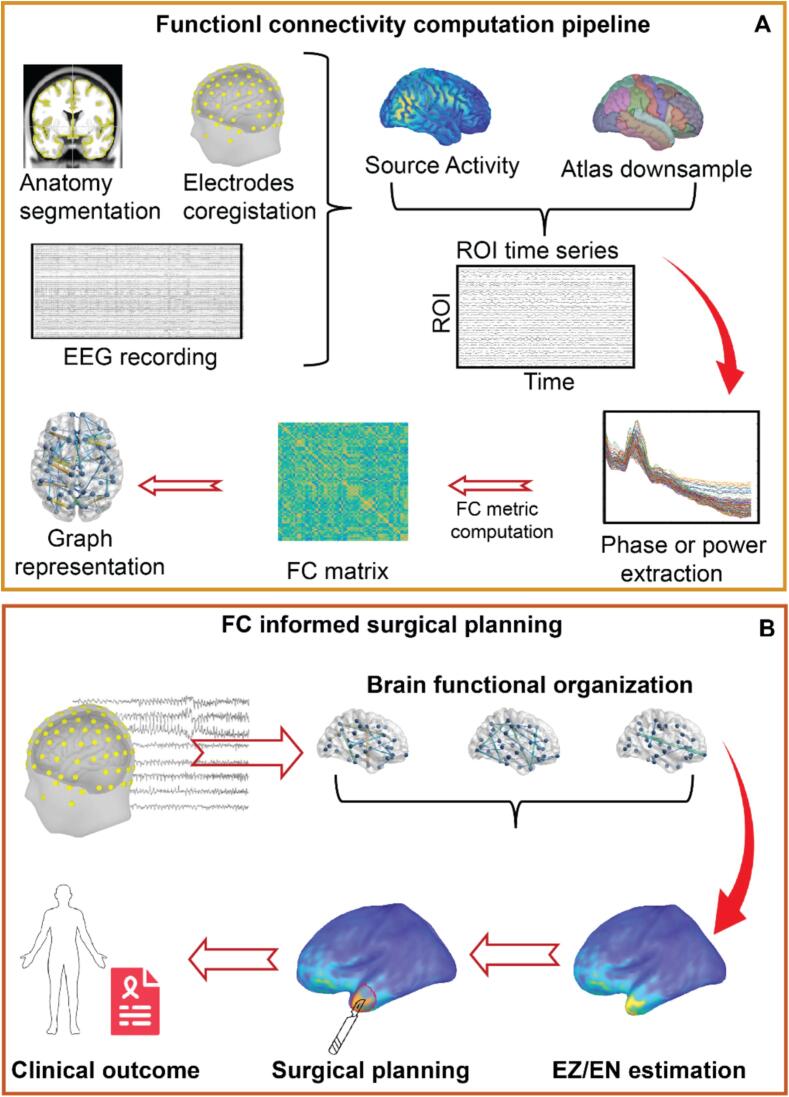
Workflow for connectivity informed surgical planning. The panel A illustrates the pipeline to compute source-derived functional connectivity from scalp EEG. Panel B shows the usage of FC-derived information to identify non-invasively the epileptogenic zone/network (EZ/EN) and to inform the surgical planning.

We divided studies based on their main goal: a) identification of the EZ in the context of surgical planning and b) prediction of surgical outcome. The first group of studies frequently used invasive EEG (i.e. stereo-EEG or electrocorticography) as ground truth for accuracy of the ESI, while the second group used post-surgical seizure outcome (i.e. Engel I) as reference. The aims and methods could overlap in some studies, which were sorted based on the prevalent approach. Studies of effective connectivity in the context of resective surgery aiming at seizure freedom and palliative surgery aiming at freedom from disabling seizures were discussed separately (Fig. 2).

#### 3.3.1 Surgical planning for curative surgery

Converging evidence identified dysfunctions of large-scale networks in epilepsy (Pittau et al., 2014). Therefore, in the context of EZ localization, the investigation of interactions across brain regions is fundamental to identifying functional nodes and links sustaining hypersynchronous activity, representing the target to be removed or disconnected (Sinha et al., 2022).

Combining FC and the directionality of information flow in the brain network (i.e., EC), recent studies maximized the localization accuracy of the EZ (Lu et al., 2012, Elshoff et al., 2013, Sohrabpour et al., 2016, Staljanssens et al., 2017a, Staljanssens et al., 2017b, Martinez-Vargas et al., 2017, Yao and Li, 2021). Specifically, the EC metric known as spectrum-weighted adaptive direct transfer function (sw-aDTF) proved to be a reliable measure for EZ localization, significantly improving the results obtained by the ESI method alone (Martinez-Vargas et al., 2017, Staljanssens et al., 2017a, Staljanssens et al., 2017b). In particular, the application of sw-aDTF resulted in equal or smaller localization errors in 91.4 % of seizures as compared to the sole use of the ESI in temporal lobe epilepsy (Staljanssens et al., 2017a). A follow-up study confirmed these findings, showing that EC analysis improved ESI localization performance by 41.5 % for SOZ estimated inside the resected zone (RZ) and by 51.4 % for SOZ estimated within 10 mm of the RZ border (Staljanssens et al., 2017b). Recently, similar results were replicated in extra-temporal lobe epilepsy (Vespa et al., 2020).

The application of graph theory can be beneficial as well (Storti et al., 2017, Corona et al., 2021, Corona et al., 2023). In directed graph, hubs with larger centrality in the network (i.e., larger betweenness centrality) have larger probability of representing the EZ, confirming that the resection zone in patients with a good outcome is characterized by a larger centrality as compared to non-epileptogenic regions (Storti et al., 2017). Further confirmation has been achieved by comparing the results of EEG-derived FC vs invasive EEG, suggesting that the implementation of FC analysis could help in the implementation of electrode grids to cover all possible critical areas (Corona et al., 2023, Li et al., 2016, Lopes et al., 2020).

Finally, a recent study implemented a machine learning (ML) approach to identify the EZ using Amplitude Envelope Correlation (see section on introduction of FC metrics) in 50 patients with drug-resistant epilepsy (Ntolkeras et al., 2024). The automatic classification reached an accuracy performance of 71 % when using epileptiform epochs for classification, which increased to 75 % when focusing on the gamma frequency band. The ML approach including brain dynamics during epileptiform activity (i.e., IEDs) showed a better performance with a lower mean distance (Euclidean distance = 38 mm) from the resected zone as compared to the ESI of the spikes (Euclidean distance = 57 mm).

#### 3.3.2 Surgical candidates identification for curative surgery

The FC analysis has been used to characterize patients with different post-surgical outcomes to extend its applicability as a prognostic instrument and aid the selection of the best candidates for surgery (Kerr and McFarlane, 2023, Rehab et al., 2024).

A favorable surgical outcome is associated with significant reorganization of brain networks, particularly in the regions surrounding or including the resected areas (Carboni et al., 2019, Iandolo et al., 2021). There is a consensus that successful surgery likely involves isolating and removing highly connected epileptic regions, disrupting the network that controls epileptic activity seizures (Zijlmans et al., 2019). Conversely, a poor surgical outcome is associated with a lack of significant changes in FC and centrality, indicating that the affected network was not effectively disrupted. Specifically, patients displaying a good surgical outcome were characterized by lower global efficiency and more localized pathological activity before surgery (Carboni et al., 2019). Similar results corroborated the relevance of global/local network configurations evidencing that a good surgical outcome was associated with increased centrality and FC in the untouched brain regions post-surgery. They suggest that non-epileptogenic regions gain centrality after a successful surgery, compensating for the removed epileptogenic focus. Conversely, unchanged or reduced centrality in these regions was associated with a poor outcome, implying insufficient network reorganization and persistence of epileptogenic activity (Iandolo et al., 2021). Further investigations leveraged artificial intelligence (i.e., machine learning) and connectivity to predict postsurgical outcomes (Pepi et al., 2022, Varatharajah et al., 2022).The interhemispheric connectivity (IHC) and interhemispheric spectral-coherence density (ISD) proved to be informative features for machine learning classification of seizure freedom (performed via artificial neural network or naïve bayes classifier). Two seminal studies used these connectivity properties (i.e., IHC and ISD) being able to predict seizure-freedom of patients undergoing hemispherotomy or anterior temporal lobectomy, with an accuracy of 80 % and 76 %, respectively (Pepi et al., 2022, Varatharajah et al., 2022).

#### 3.3.3 Surgical candidates identification for palliative surgery

Measures of connectivity were also applied in the context of palliative surgery, such as callosotomy, and in patients with generalized epilepsy (Hur and Kim, 2015, Hur and Kim, 2016, Liang et al., 2017, Liang et al., 2018, Baba et al., 2019, Uda et al., 2021, Matsuhashi et al., 2022). In fact, EC has proven to be informative also in epileptic syndromes such as Lennox-Gasteau (LGS) (Hur and Kim, 2015, Hur and Kim, 2016). Two studies analyzed generalized epileptiform discharges on pre-callosotomy EEG via direct directed transfer function (dDTF) and partial directed coherence (PDC). A subgroup of the sample showing unilateral epileptogenicity on post-callosotomy EEG underwent resective surgery. Relevantly, dDTF and PDC were able to identify concordant regions with resection areas in patients with good outcomes (Hur and Kim, 2015, Hur and Kim, 2016). Patterns of connectivity were also investigated before and after the callosotomy to characterize the target population who would benefit the most from this procedure. In Lennox-Gastaut syndrome, a good surgical outcome corresponds to: i) a shift in the functional network hubs from the paramedian to more lateral regions; ii) decreased global clustering coefficient; iii) small-worldness increase, suggesting a re-normalization of the integration-segregation balance (Liang et al., 2017, Liang et al., 2018). The effect on functional connectivity after complete or partial callosotomy has also been investigated in children with infantile spasms (Baba et al., 2019, Uda et al., 2021). The findings showed that children with seizure freedom after TCC, tended to display higher connectivity (measured with weighted phase lag index) in the delta band, whereas those with poor outcome presented stronger spectral power of fast oscillations (FOs; higher than 45 Hz) and theta connectivity. Additionally, the coupling between the power of fast frequencies (30–70 Hz) and the phase of slow activity (0.5–4 Hz) (frequency − phase coupling) proved to be sensitive to the intervention (Baba et al., 2019, Uda et al., 2021). Despite the merit of these studies in focusing on the use of connectivity as a guide for the treatment of highly refractory epilepsies, they are limited by their exploration of surgical techniques that are currently infrequently used in the patient population studied. Moreover, patients’ inclusion criteria were not always clear, leading to results that are difficult to interpret and replicate. However, they open future perspectives on the use of connectivity to guide personalized neuromodulation interventions.

#### 3.3.4 Prediction of surgical effect on neuropsychology

Multiple studies investigated the disrupted neural dynamics underlying neuropsychological impairments in patients with epilepsy (van Diessen et al., 2014, Garcia-Ramos et al., 2016, Garcia-Ramos et al., 2021, Haneef and Chiang, 2014, Holmes, 2015, Kellermann et al., 2015, Shamshiri et al., 2019, Stam, 2014). Nevertheless, there is limited evidence to support potential clinical translational application in the prediction of neuropsychological outcomes after surgery. A single study performed with this aim investigated the relationship between the developmental quotient (DQ) in children with posterior quadrantectomy and FC (Ueda et al., 2021). They observed a change in the relationship between the DQ and the FC (measured with the PLI). In the preoperative condition, a stronger long-range connectivity in the beta band was associated with better cognitive functioning (higher DQ). In the longitudinal monitoring (3 years post-surgery), a shift in the DQ-FC association was evidenced, highlighting an increase in the relevance of local (anterior) short-range connections. These findings suggest that FC may help to quantify neurodevelopmental improvements after surgery.

## 4 Discussion

The literature revision evidenced concordant findings supporting that the inclusion of functional connectivity, as compared to the sole use of ESI, improves the localization of the epileptic focus and it could inform surgical planning. The importance of functional architecture investigation in pre-surgical planning with non-invasive procedures is also supported by converging evidence from different modalities (Magnetoencephalography and functional MRI) (Aydin et al., 2020, Boerwinkle et al., 2020, Negishi et al., 2011). These findings reflect that epilepsy is a network disorder. Nevertheless, clinical translation requires more robust evidence. In the following sections of the discussion we will evidence the limitations in the applicability of this methodology that emerged across studies, and we will delineate future directions to promote solutions and improve translational application in clinical practice.

### 4.1 Limitations and open questions in FC applications

While there has been a significant effort in the research community to address this relevant topic, studies are severely affected by heterogeneity in experimental settings and data acquisition. Some precautions have to be taken when implementing FC analysis for localization purposes. The FC should be directly computed on source activation (Baillet, 2017) after the ESI of EEGs acquired with a high-density coverage. Converging findings underlined an inverse relationship between number of channels and localization error, showing that an acceptable performance can be achieved with a minimal array density of around 70 electrodes (Lu et al., 2012, Staljanssens et al., 2017a). Yet, 18 out of 26 studies used low-density EEG (from 19 to 32 electrodes) (Stoyell et al., 2021).

The EEG sampling rate has proven to be a potential source of bias, especially when considering ictal activity. In fact, when analyzing seizures at 200 vs >= 500 Hz, only a partial concordance across results has been obtained (Li et al., 2016), thus suggesting an influence of sampling rate also on source estimation.

An important open question is about the type of signal necessary to reliably perform FC for EZ localization. Only a minority of the studies performed FC on the ictal, while the majority used interictal activity, in which the source of IEDs represents a proxy for the EZ. Importantly, recent evidence suggests that source connectivity from EEG resting state activity, even without spikes, can provide relevant information regarding the epileptogenic network, enlarging the range of application of EEG (Corsi et al., 2024, Duma et al., 2023). The type of signal used is intrinsically related to the amount of data necessary to reliably reconstruct functional networks. The presence of stable connectomes with the EEG has been observed in recordings of 2 min length (Chu et al., 2012). However, recent findings suggest that larger time windows are necessary to fully capture brain dynamics, suggesting a length duration of between 5 and 10 min (Corsi et al., 2024, Liuzzi et al., 2017).

Another relevant issue, preventing the comparison of the results across studies and limiting the generalizability of FC applicability is the large variability linked to different ESI approaches as well as FC metrics. Finally, the majority of the studies are characterized by a lack of the comparison with clinical gold standard to precisely quantify the information increase in the surgical planning, derived by FC, as compared to a more standard approach based on the standard EEG, magnetic resonance imaging (MRI) and positron emission tomography (PET) examinations.

### 4.2 Directions for future investigations

The above-mentioned limitations underlie the necessity to improve methodological homogeneity across studies to promote the generalizability of FC-derived results. A first methodological, acquisition set-up oriented, recommendation is the EEG collection with a dense sensor net when recorded for localization purposes. This does not imply that FC derived by low-density EEG does not contain useful information to inform clinical practice in epilepsy. Sensor-level connectivity, obtained by a low-density montage, recently proved to be a sensitive biomarker predicting antiseizure medications efficacy (Hwang et al., 2024, Ricci et al., 2024). However, recent works provide substantial evidence of the advantage of using high density montage (HD-EEG) in practical clinical scenarios for localization purposes, highlighting the increase of the centers using this tool (Ebersole, 2024, Nucera et al., 2024, Stoyell et al., 2021).

Besides the methodological advantage of HD-EEG for source-derived FC, its contribution to surgical planning has never been directly quantified. The evaluation for pre-surgical planning in epilepsy is based on the examination of seizure semiology, neuropsychology and multimodal techniques investigating brain electrical activity (sensor-level scalp EEG), anatomical structure (MRI) and metabolic patterns (PET) (Rossi Sebastiano et al., 2020). Importantly, the majority of the reviewed studies focused on retrospective study design. However, future studies should be structured with a prospective design to quantify the added benefit of source-derived FC in the presurgical planning as compared to the standard clinical practice.

Future investigations should overcome a limitation characterizing the majority of the studies, namely a reduced sample size. In fact, a large amount of data is necessary to validate a technique for clinical practice. Public repositories and/or international consortiums may represent an important resource helping the international community to gather a sufficient amount of information. The ENIGMA-Epilepsy, a worldwide consortium focusing at the moment on neuroanatomical investigations, is a successful example of the efficacy of international data sharing providing novel insight on the neural mechanisms underlying epilepsy (Larivière et al., 2020, Larivière et al., 2022, Sisodiya et al., 2022).

To promote the translation application of FC methods in clinical practice, a fundamental milestone would require the identification of the best practice for FC computation and to define a set of measures both for source localization and connectivity to be applied for EZ identification purposes. The computation of FC is usually performed over regions of interest (ROI) of a parceled brain based on specific atlases. However, ROI signal extraction procedures have a direct impact on connectivity estimation. Recent evidence suggests dimensionality to perform reduction after connectivity computation on the full brain mesh to improve sensitivity (Brkić et al., 2023). Despite its popularity, FC is a statistical construct with an arbitrary operational definition. A recent work tried to benchmark the different FC measures concerning their capabilities to study neurophysiological networks, structure–function coupling as well as brain behavior predictions (Liu et al., 2024). The different metrics displayed differential sensitivity to the underlying mechanisms of inter-regional communication. This requires future studies to characterize all the EEG-related FC measures to benchmark the most sensitive and precise in the context of EZ localization and surgical planning.

### 4.3 Future perspectives

A useful clinical tool should be tailored to the uniqueness of the patients. Recent advancements in computational neuroscience provide the unique possibility of virtually simulating patient-specific brain dynamics via the *virtual brain twins* based on physiological and pathophysiological hypotheses, enabling individual clinical prediction and interventions (Wang et al., 2024a). In this scenario, the goodness of the prediction of simulations is obtained by comparing the FC obtained by real recordings and simulated data (Deco et al., 2013). An example of the application of *in-silico* models for clinical application is the EPINOV registered trial (Improving EPilepsy Surgery Management and progNOsis Using Virtual Epileptic Patient Software (VEP), n.d.'), a prospective study leveraging a simulative/computational approach based on solid theoretical background (Sanz-Leon et al., 2015). The Virtual Epileptic Patient (VEP) uses large-scale models, starting from the patient-specific anatomy and structural connectome, to estimate with a bayesian approach the probability of a region of being epileptogenic by comparing real and simulated ictal activity.(Hashemi et al., 2020; V. K. Jirsa et al., 2017; H. E. Wang et al., 2023). While this approach is still in the testing phase for stereo-EEG data, it represents a blueprint of upcoming scenarios generalizable to the use of EEG (Jirsa et al., 2023). Recent investigations probed the usability of *in-silico* models to simulate ictal sEEG and EEG inducted by deep-brain and non-invasive (temporal-interference) stimulations to map the epileptogenic network to provide virtualized scenarios guiding pre-surgical planning (Wang et al., 2024b). The strongest advantage of the *in-silico* models is to test virtually not only multiple hypotheses of stimulation for EZ mapping but also multiple digitized resections or disconnections scenarios, testing the effect on the electrophysiological organization using FC-related features (Jirsa et al., 2023, Kini et al., 2019). These methodological advancements represent one of the most promising future perspectives to exploit brain dynamics, and therefore functional connectivity, to inform or test the goodness of precision of predictive models to maximize non-invasive methodologies applications in epileptic patient management, thanks to the virtualization approach.

## 5 Conclusions

Overall, the present work emphasizes the potentiality of the inclusion of EEG-derived brain dynamics, in the presurgical setting as a noninvasive tool. Nonetheless, while this method has been around for decades, it still lacks clinical transition despite the potential advantages of non-invasive EZ mapping. We highlighted potential solutions that would strengthen the applicability of EEG-derived FC measurements, including: i) standardization of the acquisition protocols; ii) prospective study design to quantify information increase related to EEG-derived FC as compared to standard protocols; iii) identification and standardization of the best ESI method and FC metrics for EZ mapping; iv) application of the digital twins technology for a virtualized EZ mapping. The proposed future perspective will be able to provide not only neuroscientific advancement but also to facilitate the translation of functional connectivity to the healthcare systems.

## CRediT authorship contribution statement

**Giulia Salvatici:** Writing – review & editing, Writing – original draft, Formal analysis, Data curation. **Giovanni Pellegrino:** Writing – review & editing, Writing – original draft, Methodology, Funding acquisition, Conceptualization. **Marco Perulli:** Writing – review & editing, Writing – original draft, Methodology, Formal analysis, Data curation. **Alberto Danieli:** Writing – review & editing, Writing – original draft, Investigation. **Paolo Bonanni:** Writing – review & editing, Writing – original draft, Resources. **Gian Marco Duma:** Writing – review & editing, Writing – original draft, Visualization, Investigation, Formal analysis, Data curation, Conceptualization.

## Declaration of competing interest

The authors declare that they have no known competing financial interests or personal relationships that could have appeared to influence the work reported in this paper.

## Data Availability

No data was used for the research described in the article.
