# Supplementary material for: Electroencephalography derived connectivity informing epilepsy surgical planning: Towards clinical applications and future perspectives
Source: Neuroimage Clin. 2024 Nov 10;44:103703. doi: 10.1016/j.nicl.2024.103703 (PMC11613172; doi:10.1016/j.nicl.2024.103703)
Supplement: Supplementary Data 1 [file mmc1.docx]

The following PubMed queries were used: “epilepsy” + “surgery” + (“EEG” OR “electroencephalography”) + “network” NOT (“fMRI” OR “functional magnetic resonance imaging”) (search March 08, 2024, 549 results), “epilepsy” + “surgery” + (“EEG” OR “electroencephalography”) + “connectivity” NOT (“fMRI” OR “functional magnetic resonance imaging”) (search March 08, 2024, 271 results (119 new, 152 duplicates)) “epilepsy” + “surgery” + (“EEG” OR “electroencephalography”) + “functional organization” NOT (“fMRI” OR “functional magnetic resonance imaging”) (search March 08, 2024, 15 results (11 new, 4 duplicates)) "epilepsy" + "surgery" + ("EEG" OR "electroencephalography") + "brain dynamics" NOT ("fMRI" OR "functional magnetic resonance imaging”) (search March 08, 2024, 25 results (14 new, 11 duplicates)) , “epilepsy” + “surgery” + (“EEG” OR “electroencephalography”) + “source reconstruction” NOT (“fMRI” OR “functional magnetic resonance imaging”) (search March 08, 2024, 14 results (11 new, 3 duplicates)), “epilepsy” + “surgery” + (“EEG” OR “electroencephalography”) + “inverse model” NOT (“fMRI” OR “functional magnetic resonance imaging”) (search March 08, 2024, 3 results (3 new)), "epilepsy" + "surgery" + ("EEG" OR "electroencephalography") + "inverse solution” NOT (“fMRI” OR “functional magnetic resonance imaging”) (search March 08, 2024, 21 results (14 new, 7 duplicates)), "epilepsy" + "surgery" + ("EEG" OR "electroencephalography") + (“ESI” OR “electric source imaging”) NOT (“fMRI” OR “functional magnetic resonance imaging”) (search March 08, 2024, 86 results (65 new, 21 duplicates)), "epilepsy" + "surgery" + ("EEG" OR "electroencephalography") + "source space” NOT (“fMRI” OR “functional magnetic resonance imaging”) (search March 08, 2024, 9 results (2 new, 7 duplicates)), “epilepsy” + “surgery” + (“EEG” OR “electroencephalography”) + “resting state” NOT (“fMRI” OR “functional magnetic resonance imaging”) (search March 08, 2024, 37 results (12 new, 25 duplicates)), “epilepsy” + “surgery” + (“EEG” OR “electroencephalography”) + “resting” NOT ("fMRI " OR "functional magnetic resonance imaging”) (search March 08, 2024, 55 results (11 new, 44 duplicates)). The following query did not produce any result on PubMed: “epilepsy” + “surgery” + (“EEG” OR “electroencephalography”) + “intrinsic dynamics” NOT (“fMRI” OR “functional magnetic resonance imaging”) (search March 08, 2024).

Scopus was searched using: TITLE-ABS-KEY (("epilepsy" AND "surgery" AND ("EEG" OR "electroencephalography") AND "connectivity") AND NOT ("fMRI" OR "functional magnetic resonance imaging")) (search March 08, 2024, 229 results (68 new, 161 duplicates)) TITLE-ABS-KEY(("epilepsy" AND "surgery" AND ("EEG" OR "electroencephalography") AND "network") AND NOT ("fMRI" OR "functional magnetic resonance imaging")) (search March 08, 2024, 647 results (261 new, 386 duplicates)) TITLE-ABS-KEY(("epilepsy" AND "surgery" AND ("EEG" OR "electroencephalography") AND "functional organization") AND NOT ("fMRI" OR "functional magnetic resonance imaging")) (search March 08, 2024, 9 results (2 new, 7 duplicates)) TITLE-ABS-KEY(("epilepsy" AND "surgery" AND ("EEG" OR "electroencephalography") AND "brain dynamics") AND NOT ("fMRI" OR "functional magnetic resonance imaging")) (search March 08, 2024, 7 results (2 new, 5 duplicates)), TITLE-ABS-KEY ( ( "epilepsy" AND "surgery" AND ( "EEG" OR "electroencephalography" ) AND "source reconstruction" ) AND NOT ( "fMRI" OR "functional magnetic resonance imaging" ) ) (search March 08, 2024, 12 results (3 new, 9 duplicates)), TITLE-ABS-KEY ( ( "epilepsy" AND "surgery" AND ( "EEG" OR "electroencephalography" ) AND "inverse solution" ) AND NOT ( "fMRI" OR "functional magnetic resonance imaging" ) ) (search March 08, 2024, 20 results (5 new, 15 duplicates)), TITLE-ABS-KEY ( ( "epilepsy" AND "surgery" AND ( "EEG" OR "electroencephalography" ) AND ( "ESI" OR "electric source imaging" ) ) AND NOT ( "fMRI" OR "functional magnetic resonance imaging" ) ) (search March 08, 2024, 79 results (7 new, 72 duplicates)), TITLE-ABS-KEY ( ( "epilepsy" AND "surgery" AND ( "EEG" OR "electroencephalography" ) AND "source space" ) AND NOT ( "fMRI" OR "functional magnetic resonance imaging" ) ) (search March 08, 2024, 11 results (1 new, 10 duplicates)), TITLE-ABS-KEY ( ( "epilepsy" AND "surgery" AND ( "EEG" OR "electroencephalography" ) AND "resting" ) AND NOT ( "fMRI" OR "functional magnetic resonance imaging" ) ) (search March 08, 2024, 37 results (3 new, 34 duplicates)). The following queries returned only duplicate studies as March 08, 2024 on Scopus: TITLE-ABS-KEY ( ( "epilepsy" AND "surgery" AND ( "EEG" OR "electroencephalography" ) AND "inverse model" ) AND NOT ( "fMRI" OR "functional magnetic resonance imaging" ) ), TITLE-ABS-KEY ( ( "epilepsy" AND "surgery" AND ( "EEG" OR "electroencephalography" ) AND "resting state" ) AND NOT ( "fMRI" OR "functional magnetic resonance imaging" ) ). The following query did not produce any result on Scopus: TITLE-ABS-KEY(("epilepsy" AND "surgery" AND ("EEG" OR "electroencephalography") AND "intrinsic dynamics") AND NOT ("fMRI" OR "functional magnetic resonance imaging")) (search March 08, 2024).
